# The History and Capabilities of Herbal Simples

**Published:** 1891-08-15

**Authors:** W. T. Fernie


### The History and Capabilities of Herbal Simples.

By W. T. Fernie, M.D.

### XXXVI.—FERNS.

Ferns are called filices, from the Latin word *filum*, a thread, because of their filamentary fronds. Only some few of our native ferns possess medicinal virtues, though they may all be happily pronounced devoid of poisonous or deleterious properties. As curative simples I shall briefly consider the common Male and female ferns, the Royal fern, the Hart's Tongue, the Maidenhair, the common Polypody, the Spleenwort, and the Wall Rue. Each of these owes its respective usefulness chiefly to the tannin which it contains; whilst those more specially endowed with healing powers yield also a particular chemical acid called filicic, which is fatal to worms. Generically, the term "fern" has been referred to the word "feather," because of the pinnate leaves; or to *farr*, a bullock, from the use of the plant as litter for cattle; or thirdly, to *fearh*, a pig, for a like reason. Matthiolus, speaking of the male and female ferns, says, "Utriusque radice sues pinguescunt;" and in an old charter, A.D. 855, the pasturage of swine is named *fearnleswe*. In some parts of England ferns at large are known as Devil's brushes; and to bite off close to the ground the first fern which appears in the spring is said in Cornwall to cure toothache, whilst preventing its return during the remainder of the year. Bracken, or Brakes, which grows throughout England more freely than any other of the tribe, is the *Filix fœmina*, or common female fern. It is also named botanically *pteris aquilina*, because the figure which appears in its succulent stem when cut obliquely across at the base, has been thought to resemble a spread eagle, therefore Linnæus termed it *aquilina*. Some call it, for the same reason, King Charles in the oak tree; and in Scotland this is said to be an impression of the devil's foot. Again, witches are reputed to detest this fern because it bears on its root the Greek letter X, which is the initial of Christos. The Bracken is strictly confined to uncultivated ground and to waste places. As Horace testified in Roman days, "Neglectis urenda Filix innascitur agris." In the seventeenth century it was customary to set fire to growing Brakes, believing that this would produce rain. A like custom of "firing the bracken" still prevails to-day on the Devonshire moors. By an official letter the Earl of Pembroke admonished the High Sheriff of Stafford to forbear the burning of ferns during a visit of King Charles I., "as his Majesty desired that the country and himself may enjoy fair weather as long as he should remain in those parts." The bracken contains much potash, and its ashes were formerly used in the manufacture of soap. The young tops of the plant are boiled in Hampshire for hogs' food; and the peculiar flavour of Hampshire bacon has been attributed to this custom. The root contains much starch, and is employed medicinally. For thigh aches (sciatica) says an old writer, smoke the legs thoroughly with fern braken. In northern climates a coarse kind of bread is made from the roots of the brake fern, whilst in the south the young shoots are often sold in bundles as a salad. Persons who prepare leather of the kid or chamois, employ this and other ferns in dressing the skin because of their astringency.

The common male fern, Filix Mas or Shield Fern, grows abundantly in all parts of Great Britain, and has been known from the times of Dioscorides and Theophrastus as a specific remedy for intestinal worms, particularly the tape-worm. For medicinal purposes, the green part of the rhizome is kept and dried. This is then powdered, and its oleo-resin is extracted by ether. The green fixed oil thus obtained, which is poisonous to worms, consists of the glycerides of filocylic and filosmylic acids, with tannin, starch, gum, and sugar.

When the vigour of the parasite has been first reduced by a low diet for a couple of days, from thirty to ninety grains of the powdered root, or from fifteen to thirty drops of the oleo-resin should be given as a dose whilst fasting. A knowledge of this remedy had become lost until it was purchased by the French king under the advice of his principal physicians, in 1775, from a surgeon's widow in Switzerland, who employed it as a secret mode of cure with infallible success. A purgative assists the action of the plant, though it is independently quite efficacious. Gerarde said "the root of the male fern being taken to the weight of half-an-ounce driveth forth long flat worms out of the belly, as Dioscorides writeth." Pliny extolled its virtues as a worm killer, and advised its use for gout. Also the expressed mucilaginous juice has been much lauded as an outward application to burns. In 1825, an ethereal liquid extract was made from the male fern, but it was scarcely used as a vermifuge until 1851. A medicinal tincture is now prepared from the root-stock with proof spirit in the autumn when the fronds are dying. The young shoots and curled leaves of this fern are often eaten like asparagus, whilst the fronds make an excellent litter for horses and cattle. The ashes when burnt contain much vegetable alkali, which has been freely used in making glass. Mixed with water, so as to make fern balls, and heated, they act as a ley for scouring linen. The seed of this and of some other species of fern is so minute—one frond producing more than a million—as not to be visible to the naked eye. Hence, on the doctrine of Signatures, the plant has been thought to confer invisibility. So Shakespeare says—Henry IV., Act II., Sc. I—"We have the receipt of fern seed; we walk invisible." It used to be customary in England to "watch the fern" on Midsummer Eve, a time of special importance in Elfland. The plant then became mysterious, and put forth at dusk a small blue flower, which soon disappeared, and the wonderful seed, quickly ripening, was supposed to fall from the plant at midnight. It was collected in a white napkin, being known as wish-seed, and anyone carrying it about was said to be able to discover treasures hid in the earth, or to unlock secret fastenings, however difficult. The sap of this fern was thought to confer on anyone then drinking it the gift of perpetual youth. Modern writers give the name of Lady Fern, not to the bracken, but to the *Asplenium Filix fœmina*, because of its delicate and graceful foliage. "The powder thereof freely beaten healeth the galled necks of oxen and other cattell." The female fern is branched, whilst the male has only one main rib. The latter is most powerful as an antiseptic and an astringent. Female ferns are more viscid, mucilaginous, and stimulating to the kidneys.

(*To be continued.*)

